# Inhibiting cholinergic signalling in the cerebellar interpositus nucleus impairs motor behaviour

**DOI:** 10.1111/ejn.16066

**Published:** 2023-07-16

**Authors:** Jasmine Pickford, Cristiana I. Iosif, Zafar I. Bashir, Richard Apps

**Affiliations:** School of Physiology, Pharmacology and Neuroscience, University of Bristol, Bristol, UK

**Keywords:** acetylcholine, cerebellum, feeding behaviour, motor activity, motor skills, reward

## Abstract

The role of neuromodulators in the cerebellum is not well understood. In particular, the behavioural significance of the cholinergic system in the cerebellum is unknown. To investigate the importance of cerebellar cholinergic signalling in behaviour, we infused acetylcholine receptor antagonists, scopolamine and mecamylamine, bilaterally into the rat cerebellum (centred on interpositus nucleus) and observed the motor effects through a battery of behavioural tests. These tests included unrewarded behaviour during open field exploration and a horizontal ladder walking task and reward-based beam walking and pellet reaching tasks. Infusion of a mix of the antagonists did not impair motor learning in the horizontal ladder walking or the reaching task but reduced spontaneous movement during open field exploration, impaired coordination during beam walking and ladder walking, led to fewer reaches in the pellet reaching task, slowed goal-directed reaching behaviour and reduced reward pellet consumption in a free access to food task. Infusion of the muscarinic antagonist scopolamine on its own resulted in deficits in motor performance and a reduction in the number of reward pellets consumed in the free access to food task. By contrast, infusion of the nicotinic antagonist mecamylamine on its own had no significant effect on any task, except beam walking traversal time, which was reduced. Together, these data suggest that acetylcholine in the cerebellar interpositus nucleus is important for the execution and coordination of voluntary movements mainly via muscarinic receptor signalling, especially in relation to reward-related behaviour.

## Abbreviations

ANOVA: analysis of variance
BORIS: Behavioral Observation Research Interactive Software
ChAT: choline acetyltransferase
drug: mix of cholinergic antagonists scopolamine and mecamylamine
fps: frames per second
LED: light-emitting diode
Mec: mecamylamine
Phys: physostigmine
PPN: pedunculopontine nucleus
Scop: scopolamine.

## 1 Introduction

Cerebellar contributions to motor control, including the coordination of voluntary movements, balance, posture and locomotion, have long been known (Holmes, 1917; Ito, 1984, 2006; Morton & Bastian, 2004; Thach et al., 1992). There is also increasing recognition that cerebellar contributions to behaviour extend to higher-order processes including cognition (Chabrol et al., 2019; Gao et al., 2018; Grafman et al., 1992), attention (Allen et al., 1997) and reward processing (Heffley & Hull, 2019; Kostadinov et al., 2019; Larry et al., 2019; Wagner et al., 2017).

As well as major glutamatergic inputs in the form of mossy fibre and climbing fibre afferents, the cerebellum receives neuromodulatory projections (Carlson et al., 2021; Schweighofer et al., 2004) about which much less is known. In particular, extensive cholinergic inputs have been described anatomically (Jaarsma et al., 1997; Woolf & Butcher, 1989). Given the role of acetylcholine in other brain regions in learning and memory, modulation of brain states, sensory processing, attention and reward (for reviews, see Picciotto et al., 2012; Colangelo et al., 2019), it is important to understand the role acetylcholine may play in cerebellar contributions to behaviour.

Pathway tracing studies combined with choline acetyltransferase (ChAT) staining have shown ChAT-immunoreactive fibres in the cerebellum arising from a number of brainstem nuclei, notably the pedunculopontine nucleus (PPN), but also the laterodorsal tegmental nucleus, lateral paragigantocellular nucleus, dorsal raphe nucleus, nucleus raphe obscurus, vestibular nuclei and spinal trigeminal subnucleus interpolaris (Jaarsma et al., 1997; Woolf & Butcher, 1989). The functions of these nuclei span sensory (Pierret et al., 2000), motor (Brudzynski et al., 1988; Pahapill & Lozano, 2000), reward/motivational (Coimbra et al., 2019; Hong & Hikosaka, 2014; Nakamura, 2013; Skvortsova et al., 2021), homeostatic (Dergacheva et al., 2010) and sleep processes (Depuy et al., 2011; Kroeger et al., 2017; McCann et al., 1989; Sirieix et al., 2012).

The potential contributions of the cerebellar cholinergic system to motor control remain unknown (Zhang et al., 2016), but given the function of the cholinergic system in other parts of the central nervous system, we hypothesise it is important in reward-related motor behaviour. The interpositus nucleus contains a dense plexus of cholinergic inputs (Jaarsma et al., 1997), and many studies have investigated the critical role of this region in limb control. There is a somatotopic representation of the forelimbs and hindlimbs in the anterior interpositus nucleus (Garwicz & Ekerot, 1994; van Kan et al., 1993), and microstimulation within interpositus anterior results in multisegmental movement of the ipsilateral forelimb (Ekerot et al., 1995). The activity of interpositus neurons is synchronised with the step cycle during locomotion (Armstrong & Edgley, 1984), and perturbation of interpositus activity results in disrupted locomotor behaviour (Sarnaik & Raman, 2018). Interpositus activity is also modulated during reach to grasp movements (van Kan et al., 1994), and inhibiting interpositus neurons has been shown to alter reach kinematics (Becker & Person, 2019).

As a first step towards characterising the functional significance of cholinergic signalling in the cerebellum, we locally infused acetylcholine receptor antagonists into the cerebellum, centred on the interpositus nucleus, and investigated subsequent effects on exploratory, locomotor and reaching-based behaviours. In the presence of the antagonists, general locomotor activity was decreased, and skilled motor behaviours were generally slower and less accurate, although motor learning in the horizontal ladder walking and pellet reaching tasks was unaffected. There was also a reduction in the consumption of food rewards. Together, the current data show that acetylcholine supports cerebellar processing to enhance the implementation, coordination and speed of limb movements but raise the possibility that there may also be non-motor contributions such as attentional, motivational and/or appetitive processes, which indirectly influence motor performance.

## 2 Materials and Methods

### 2.1 Ethical approval

All animal procedures were performed in accordance with the UK Animals (Scientific Procedures) Act of 1986 and were approved by the University of Bristol Animal Welfare and Ethical Review Body and UK Home Office (PPL number PA26B438F).

### 2.2 Experimental animals

Experiments were conducted in adult male Lister Hooded rats (*n* = 78, 330–450 g, Envigo, UK), which were housed in pairs under a 12/12-h reverse light/dark cycle (light phase 20:15–08:15); experiments were performed during the dark phase. Standard laboratory chow was available ad libitum prior to and during recovery from surgical procedures, and after recovery animals were fed approximately 18 g of chow per day, in addition to food rewards obtained in some of the behavioural tasks. Weights of the animals were monitored to ensure they did not drop below 90% of the normal growth curve. Water was available ad libitum.

### 2.3 Surgical procedures

Rats were anaesthetised with ketamine/medetomidine (initial dose 50/0.3 mg kg^−1^ intraperitoneally, Vetalar/Domitor). Depth of anaesthesia was monitored regularly by testing the hindpaw withdrawal reflex and supplementary doses of ketamine/medetomidine were given as required. Stainless steel bilateral guide cannulae (26 G, Plastics One, Bilaney, UK) were implanted through burr holes in the skull (11.16 mm caudal, ±2.5 mm lateral and 4.9 mm deep relative to bregma) and secured to the skull with skull screws and dental acrylic. Dummy cannulae (26 G, Plastics One, Bilaney, UK) secured with dust caps kept the guide cannulae patent between infusions. At the end of each surgery, rats were given a medetomidine antidote (atipamezole, 0.1 mg intraperitoneally), long-lasting analgesic (Metacam, 1 mg kg^−1^ subcutaneously) and saline (10 ml kg^−1^ subcutaneously). Rats were singly housed for 7 days following surgery and then returned to their original pairings. Infusions were performed no sooner than 7 days following surgery.

### 2.4 Infusions

All pharmacological agents were dissolved in 0.9% saline. Five different types of infusion were carried out: (i) a mix of the muscarinic antagonist scopolamine hydrobromide (26 mM, Tocris) and nicotinic antagonist mecamylamine hydrochloride (50 μM, Tocris), hereon termed ‘drug’; (ii) scopolamine alone (26 mM, Tocris); (iii) mecamylamine alone (50 μM, Tocris); (iv) physostigmine (5 mM, Tocris); and (v) 0.9% saline as a vehicle control. The experimenter was blinded to which solution was being infused until after analysis had taken place. Infusions were delivered through double 33-G infusion needles (Plastics One, Bilaney, UK) inserted into the guide cannulae, with 1 mm protrusion from the end of the guide cannulae. Infusions of 500 nL were delivered over 1 min into each side of the cerebellum, and the infusion cannula left in situ for a further 3 min before the dummy cannula was replaced. Behavioural testing began 15 min after the start of the infusion unless otherwise stated.

### 2.5 Behavioural testing

All behavioural testing was performed in white light (approximately 140 lx). For tasks involving food reward, 45 mg purified rodent tablets were used (TestDiet LabTab AIN-76, 5TUL, catalogue number 1811155).

### 2.6 Open field

Open field exploration was used to test how spontaneous behaviour and anxiety levels in a novel environment were affected by drug infusion. Rats were placed in the centre of a circular arena (90 cm diameter, 51 cm height) with a black matte plastic floor and allowed to freely explore for 10 min, following infusion of either saline or drug. Behaviour was monitored by an overhead webcam at 30 frames per second (fps). Post hoc, rats were tracked using DeepLabCut software (Mathis et al., 2018) using the implanted cannula on the head to track position. Distance travelled, amount of time spent in the centre of the arena (central two-thirds of open field by diameter) and number and duration of periods in which animals were stationary were calculated using custom MATLAB scripts. The number of rearing events were counted manually.

### 2.7 Elevated plus maze

The elevated plus maze tests spontaneous exploration and is used to assess how anxiety was affected by drug infusion. Rats were placed in the centre of a plus-shaped maze (110 cm wide) elevated 105 cm from floor level. The maze had two open arms and two walled arms (walls 32.5 cm high), in which rats were allowed to freely explore for 5 min following infusion of either saline or drug. Video recordings were obtained via an overhead webcam at 30 fps. The amount of time rats spent in each portion of the maze was recorded using BORIS software (Friard & Gamba, 2016).

### 2.8 Beam walking

The beam walking task was used to assess how locomotion, while maintaining balance, was affected by drug infusion in trained animals. Rats were habituated to a dark, sheltered box at the end of a narrow beam, which contained reward pellets (Figure 3ai). Rats were then trained to traverse a beam (164 cm long, 92 cm high from floor) to reach the end box to obtain five reward pellets. After allowing time to consume the reward, rats were placed back at the start of the beam for repeated trials. As rats successfully learned to cross the beam, the width of the beam was reduced from 6 to 4 cm and then to 2 cm. Experiments to test performance following infusions were carried out on the 2 cm beam. Rats were infused with saline or pharmacological agent in a counterbalanced manner and video recorded while performing six crossings of the beam, in which animals were motivated by reward pellets. Video recordings were obtained from side and bottom-up views of the beam at 30 fps. Traversal time, the time taken from starting to walk across the beam to reaching the end box, and number of foot slips were recorded in BORIS, and the median value obtained from the six trials taken from each subject was used for group analysis.

### 2.9 Horizontal ladder walking

Rats were trained on a horizontal ladder walking task (Metz & Whishaw, 2002, 2009) in which both performance and learning following infusions were assessed. Two Perspex panels (100 cm long, 20 cm high) formed the side walls of the ladder, and holes 1 cm from the bottom of each panel, at 1 cm intervals, allowed 3 mm diameter metal rungs to be inserted with different spacing between them to form the rungs of the ladder (Figure 3bi). The ladder was positioned 45 cm above the floor. To ensure rats travelled in one direction, the width of the ladder was adjusted to prevent animals turning around. The ladder was rested on a cage at each end—one of which was the rat’s home cage and the other an identical but empty cage. The home cage provided sufficient motivation for rats to cross the ladder without need for food rewards.

Rats were habituated to the home cage position in the testing room with their cage mate for 5 min. During subsequent training and testing, the cage mate was placed in a separate cage a short distance away from the home cage. This prevented the cage mate climbing onto the ladder but kept them in close proximity so as to provide motivation for the rat being tested to go to the home cage. During training, rungs were spaced at 2 cm. Each rat was placed at the start of the ladder and allowed to traverse to reach their home cage for five consecutive trials on 3 days. On the first test day, the rungs were arranged 3 cm apart, and rats were infused with saline or drug before being presented with the new rung configuration and subsequently tested on eight consecutive trials. The following day another 8 trials were completed on the 3 cm rung spacing without any infusions. After an interval of 1–4 weeks, they were again given saline or drug before testing with 3 cm rung spacing to examine the effects of the drug on the acquired task (following 2 further habitation days). Trials were video recorded at 60 fps.

Individual steps were scored frame by frame and recorded in BORIS. Scoring is described in detail by Metz and Whishaw (2002, 2009) and summarised in Table 1. Higher average scores denote better overall performance. An error was defined as a step with a score of 0, 1 or 2. For test experiments, scores per animal were based on an average of eight ladder crossings. Traversal time, the time in seconds taken from being placed on the ladder to entering the home cage, was also recorded.

### 2.10 Pellet reaching

A pellet reaching task was used to assess effects of drug infusion on learning and performance of a skilled movement motivated by food reward. The task design was based on Torres-Espín et al. (2018) who developed a motorised pellet dispenser. The training enclosure was made from clear acrylic (0.7 cm thick, 40 cm long, 14 cm wide and 45 cm high). The floor of the enclosure contained a grid of holes (6 mm diameter), which prevented rats from eating pellets dropped during the task. At the front of the enclosure was a vertical slit (1 cm wide, 10 cm tall), in front of which the pellet dispenser was placed. Pellets were dispensed 3 cm higher than the floor and at 2 cm from the inner wall of the enclosure (Figure 4a). To restrict rats to reaching with their preferred limb (see below), the dispenser was offset from the centre in line with the edge of the slit opposite the preferred limb. At the back of the box (10 cm from the back, 2.5 cm from gridded floor), an infrared LED and receiver were located. Together with the pellet dispenser, these were connected to an Arduino Uno, which triggered the release of a pellet when the LED beam was broken. This arrangement meant that the presentation of pellets was controlled by the rats shuttling to the back of the box and therefore allowed the main training stages of the task to be fully automated.

Following habituation to the enclosure (10 min), on subsequent days, reward pellets were manually placed on the edge of the pellet dispenser (10 min on 4 consecutive days). As rats learned to retrieve the pellet, the position of the pellet was moved progressively further towards the location where pellets would be delivered by the dispenser. This initial training period also allowed the experimenter to determine the preferred reaching limb of each rat. Once rats were willing to reach for pellets at the dispensed location, automated training sessions began. Rats were placed in the enclosure for 10 min per day and allowed to perform as many reaches as they initiated (based on LED beam breaks by shuttling to the back of the apparatus between reaches). For half of the animals (*n* = 7), sessions were video recorded at 30 fps. Individual reaches were categorised as (i) successful (rat retrieved and consumed the pellet), (ii) successful first attempt (retrieved and consumed pellet on first reach) or (iii) fail (rat knocked or dropped the pellet). In the other half of rats (*n* = 7), video was recorded at 160 fps. In these experiments, reaches were scored frame-by-frame in BORIS to assess outward reach duration (time from paw lift off to contact with pellet or dispenser) and the end point of the reach (the distance from the outer edge of the enclosure at the end of the outward reach).

For experiments examining performance on the task, rats were trained until their performance plateaued at a success rate of at least 60% over 3 days. Rats were then infused with saline or pharmacological agent, in a counterbalanced manner, before a 10 min test session. For experiments examining learning, rats were infused with saline or drug immediately after each daily training session, from the first to the tenth automated training session. Rats underwent a further 10 days of training without infusions.

### 2.11 Free access to reward pellets

In addition to being tested in the open field (see above) a sample of rats were re-exposed to the same circular arena. They were habituated to the environment for 5 min on two occasions on the following day and presented with reward pellets in a small bowl in the centre of the arena to reduce anxiety when entering the centre of the open environment. On test days (3 and 6 days later), rats were infused with saline or pharmacological agent in a counterbalanced manner before being placed into the arena, which contained at its centre a small bowl filled with reward pellets. The pellets were weighed before and after the session, and the approximate number of pellets consumed was calculated based on the difference in weight.

### 2.12 Terminal perfusion and histological processing

At the end of every experiment, rats were deeply anaesthetised with sodium pentobarbital (Euthatal, 200 mg/mL) and transcardially perfused with 0.9% saline followed by 4% paraformaldehyde in 0.1 M phosphate buffer. In all cases, brains were extracted, post-fixed in 4% paraformaldehyde overnight, then cryoprotected in 30% sucrose solution. To verify cannula position, the cerebellum was cut into 50 μm sagittal sections and visualised using an epifluorescence microscope fitted with bright-field illumination (Zeiss Axioskop 2 Plus, 2.5×/0.075 lens). To estimate spread of infusate, a sample of rats (*n* = 4) were infused with fluorescein sodium salt (Sigma-Aldrich) in 0.9% saline (10 mg/mL, 500 nL over 1 min, infusion cannula in place for further 3 min) approximately 15 min before transcardial perfusion. Fluorescein has a similar molecular weight to the drugs infused (fluorescein 376.3 MW, scopolamine 384.3 MW, mecamylamine 203.8 MW). Infusate spread was successfully visualised post-mortem in two of these rats.

### 2.13 Statistical analysis

Data are presented as mean ± standard error of the mean. All statistical analyses were performed using IBM SPSS Statistics for Windows (Version 24.0. Armonk, NY: IBM Corp.). Where rats underwent infusion of saline and drug in the same experimental paradigm, a paired *t*-test was used. Where animals were split into separate saline and drug groups, an independent *t*-test was used. Pearson’s correlation analysis was performed when assessing the relationship between reach duration and endpoint. For learning experiments, a mixed ANOVA was used as animals were split into two groups (saline and drug), with trial/training session as the within-subject factor and treatment group the between-subject factor. Data were assessed to ensure they met the assumptions of the statistical test, and appropriate corrections for data violating normality or sphericity assumptions were made where necessary (detailed as appropriate in Results). For ANOVA data violating sphericity, the uncorrected degrees of freedom with the corrected *P*-value are reported (together with ε). A *P*-value of less than 0.05 was considered statistically significant.

## 3 Results

### 3.1 Infusions were centred on the interpositus nuclei of the cerebellum

Rats were implanted with bilateral indwelling cannulae targeting both the left and right interpositus nuclei of the cerebellum. The cannulae allowed infusion of a cocktail of the acetylcholine receptor antagonists scopolamine (26 mM) and mecamylamine (50 μM) to block both muscarinic and nicotinic signalling, respectively (‘drug’). In a subset of experiments, the cholinergic antagonists were infused either separately or with the cholinesterase inhibitor physostigmine (5 mM). In all cases, drugs were dissolved in 0.9% saline (vehicle).

**FIGURE 1:**
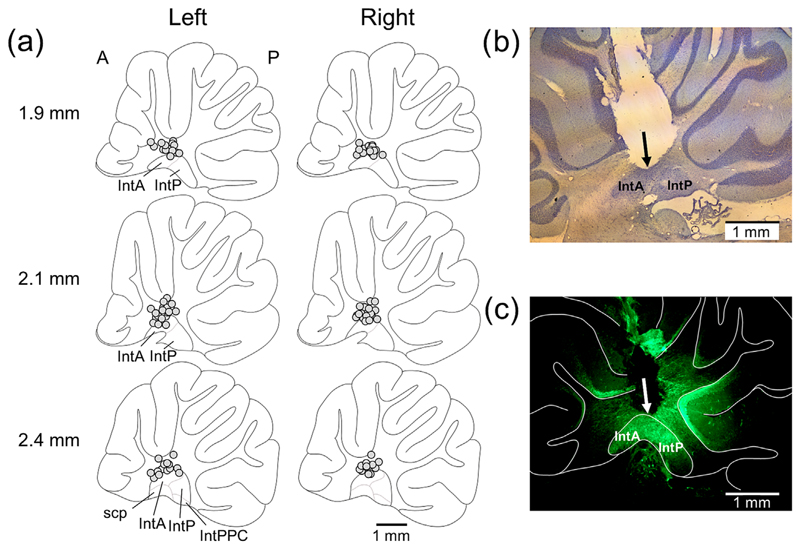
Histological verification of cannula placement and infusion spread. (a) Approximate cannula tip locations depicted on sagittal cerebellar outlines at different medio-lateral distances from bregma for left and right side of cerebellum (from Paxinos & Watson, 2007). Each animal is represented by a separate circle. (b) Example micrograph of sagittal cerebellar section showing cannula location; arrow indicates cannula tip. (c) Example of fluorescein spread (500 nL) in sagittal cerebellar section; arrow indicates cannula tip. A, anterior; IntA, interpositus anterior; IntP, interpositus anterior; IntPPC, interpositus posterior parvicellular part; P, posterior; scp, superior cerebellar peduncle.

Post-mortem histological reconstruction of the location of the cannulae showed that in every case, the tip was in the white matter just above or in the dorsal part of the interpositus nucleus (Figure 1a,b). An estimate of infusion spread was determined post-mortem in two rats using fluorescein (see Section 2.12 for details). In both cases, the infusion spread approximately 1.3 mm from the cannula tip (Figure 1c). The histology therefore suggests that all infusions were centred on the interpositus nuclei, but there was likely to be some spread to adjacent nuclear regions and overlying deep cerebellar cortex. No spread outside of the cerebellum was detected.

### 3.2 Infusion of cholinergic antagonists into the interpositus nuclei reduces non-goal-directed locomotion

**FIGURE 2:**
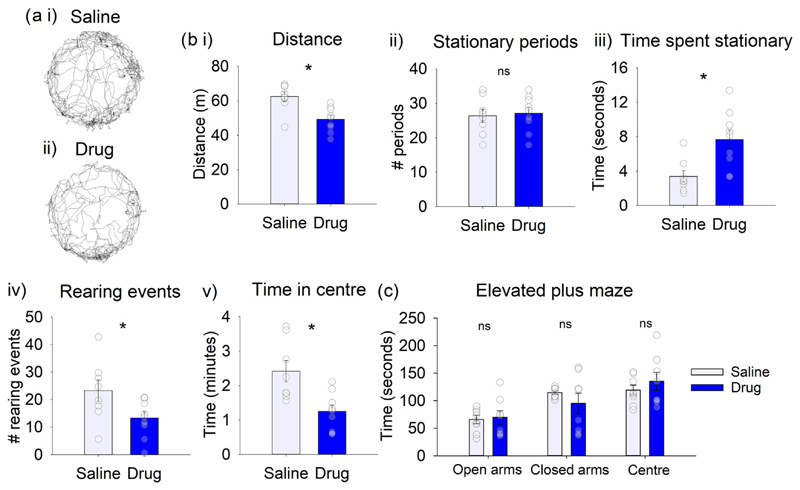
Infusion of cholinergic antagonists into the interpositus nuclei of the cerebellum decreased motor behaviour in a novel open field arena. (a) Example trajectories of exploration following infusion of either (i) saline or (ii) drug (scopolamine and mecamylamine) into the interpositus nuclei. (b) (i) total distance travelled, (ii) number of stationary periods, (iii) duration of stationary periods, (iv) number of rearing events and (v) time spend in centre of the arena following infusion (*n* = 8 saline, *n* = 9 drug). (c) Time spent in each section of the elevated plus maze following infusion (*n* = 8 saline, *n* = 8 drug). *P < 0.05; ns, not statistically significant.

To investigate the role of cerebellar cholinergic signalling in general locomotor behaviour, rats received an infusion of drug (*n* = 9) or vehicle (saline, *n* = 8) before being placed into a novel open field arena for 10 min (Figure 2a). Rats that received a drug infusion travelled significantly less distance than those that received a saline infusion (Figure 2bi, *t*_15_ = 3.596, *P* = 0.003). There was no significant difference between saline and drug infusions in the number of periods rats were stationary in the arena (Figure 2bii, *t*_15_ = −0.284, *P* = 0.780). However, those receiving the drug treatment were stationary for longer periods of time (Figure 2biii, *t*_15_ = −3.190, *P* = 0.006). Following drug infusion, animals also showed a significant reduction in rearing activity (Figure 2biv, *t*_15_ = 2.267, *P* = 0.039).

Rats with drug infusion spent less time in the centre of the arena compared to those infused with saline (Figure 2bv, *t*_15_ = 3.337, *P* = 0.005). This difference may be related to either a general reduction in locomotor activity and/or increased anxiety. To investigate the latter, rats were also tested on the elevated plus maze (*n* = 8 rats per group). There was no significant difference in time spent in any sector of the maze between animals receiving saline or drug infusions (Figure 2c; open arms: *t*_14_ = −0.260, *P* = 0.799; closed arms: *t*_14_ = 1.047, *P* = 0.328, corrected as equal variances not assumed; centre: *t*_14_ = −0.856, *P* = 0.406). Taken together, these results therefore suggest that, in the open field, the main effect of infusion of a mix of muscarinic and nicotinic antagonists into the interpositus nuclei is to reduce spontaneous motor activity (locomotion and rearing), rather than having any major effect on anxiety levels.

### 3.3 Infusion of cholinergic antagonists into the interpositus nuclei impairs goal-directed locomotion

**FIGURE 3:**
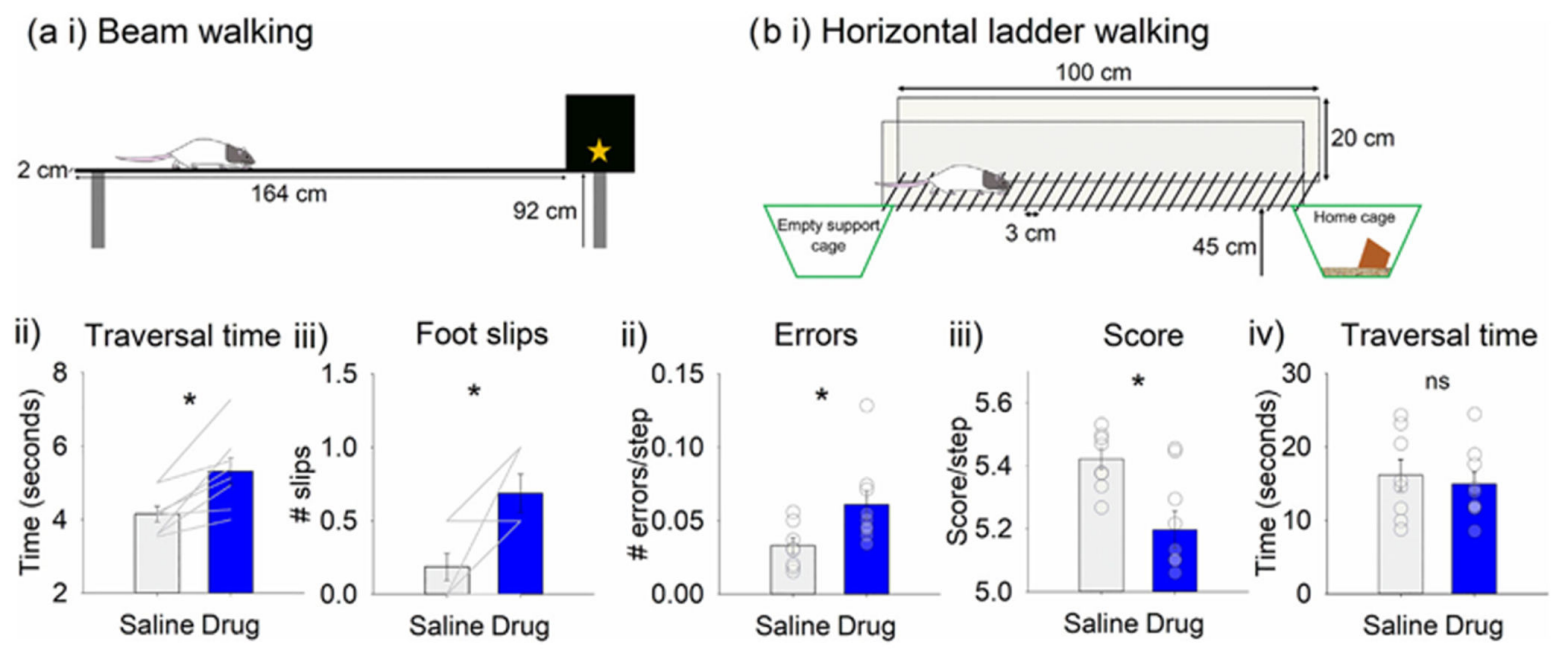
Infusion of cholinergic antagonists into the interpositus nuclei of the cerebellum impaired performance on the beam walking and horizontal ladder walking tasks. (a) Beam walking: (i) task schematic, (ii) traversal time and (iii) number of foot slips following infusion of saline or drug (scopolamine and mecamylamine, *n* = 8). (b) Horizontal ladder walking: (i) task schematic, (ii) average number of errors per step, (iii) average score per step and (iv) traversal time following infusion (saline *n* = 8, drug *n* = 9). *P < 0.05; ns, not statistically significant.

To further investigate effects of cholinergic antagonist infusion into the interpositus nuclei on locomotion, a beam walking and a horizontal ladder walking task were used. In the beam walking task, rats were required to traverse a narrow beam to reach a goal box in which there was a food reward (Figure 3ai, *n* = 8 rats). It took significantly longer for rats with a drug infusion to traverse the beam compared to rats with saline infusion (Figure 3aii, *t*_7_ = −4.098, *P* = 0.005). The drug infusion also resulted in significantly more foot slips (Figure 3aiii, *t*_7_ = −3.742, *P* = 0.007).

In the horizontal ladder walking task, rats were required to cross the rungs of a horizontal ladder in order to reach their home cage (see Section 2.9 for details; Figure 3bi, saline *n* = 8, drug *n* = 9). Rats with a drug infusion made significantly more stepping errors when crossing the ladder than those infused with saline (Figure 3bii, *t*_15_ = −2.484, *P* = 0.025). As a result of the increase in errors there was a decreased average score per step (Figure 3biii, *t*_15_ = 3.214, *P* = 0.006). However, there was no significant difference between the two groups in the time taken to traverse the ladder (Figure 3biv, *t*_15_ = 0.461, *P* = 0.652). In summary, there was a significant slowing of traversal time in the beam but not the ladder task (possible reasons for this are considered in Section 4). However, in both tasks, localised infusion of a mix of muscarinic and nicotinic antagonists into the interpositus nuclei resulted in a significant increase in stepping errors.

### 3.4 Infusion of cholinergic antagonists into the interpositus nuclei slows forelimb reaching

**FIGURE 4:**
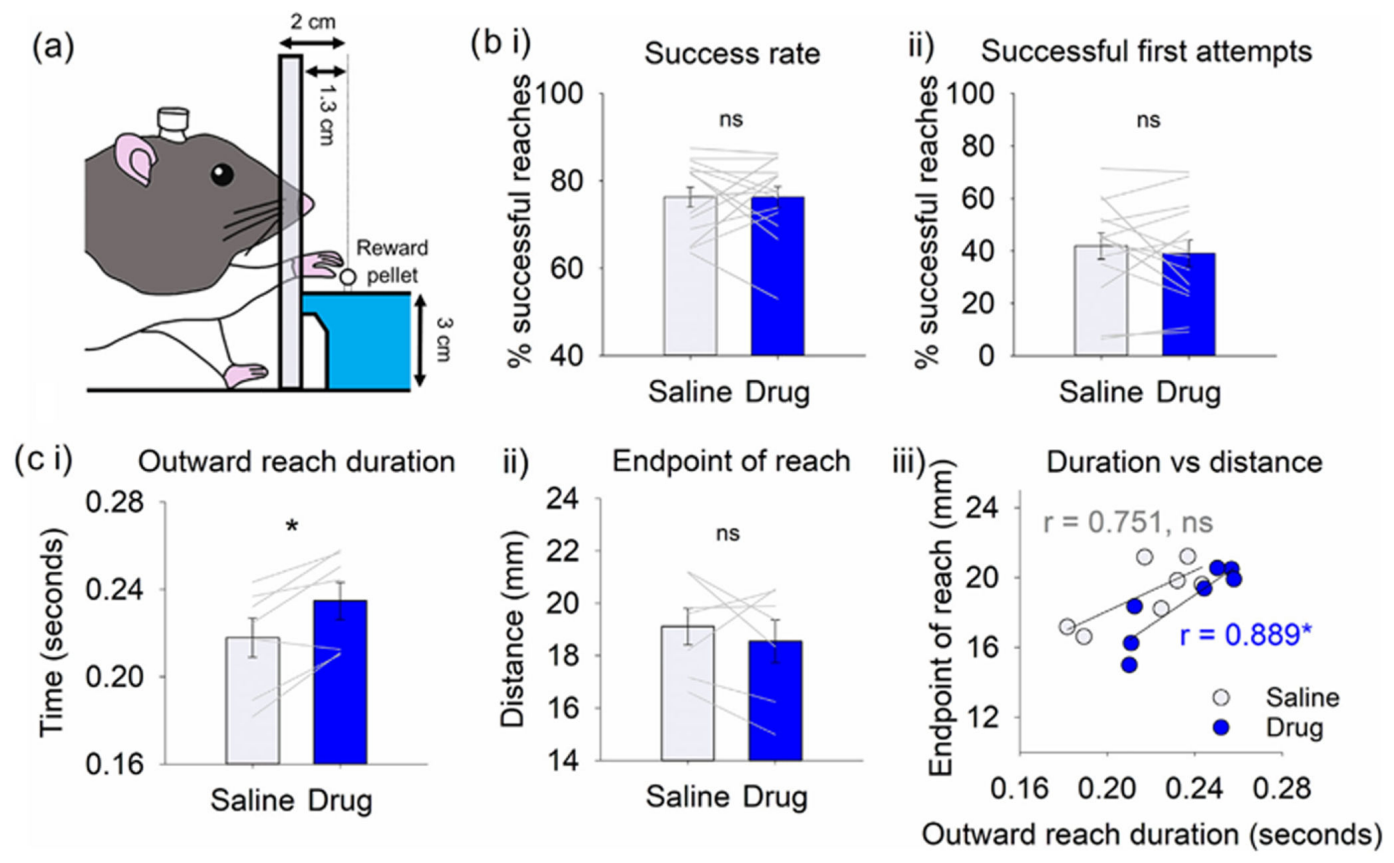
Infusion of cholinergic antagonists into the interpositus nuclei of the cerebellum slowed reaching activity in a pellet reaching task. (a) Schematic of single pellet reaching task. (b) Proportion of reaches in which a pellet was retrieved on (i) any attempt and (ii) on the first attempt following infusion of saline or drug (scopolamine and mecamylamine, *n* = 14). (c) In a subset of rats (*n* = 7), (i) outward reach duration and (ii) end position of the reach were measured; (iii) correlation of outward reach duration and end position following infusion. *P < 0.05; ns, not statistically significant.

The single pellet reaching task was used to investigate effects of cholinergic antagonists in the interpositus nuclei on a skilled, reach-to-grasp movement motivated by food reward (Figure 4a, *n* = 14). There were no significant differences between saline and drug groups when examining the proportion of successful reaches throughout the 10 min session (Figure 4bi, *t*_13_ = 0.007, *P* = 0.995; see Section 2.10). The proportion of reaches in which the rats retrieved the reward pellet on their first attempt was also not significantly different (Figure 4bii, *t*_13_ = 0.723, *P* = 0.483). Together, these results therefore suggest that the drug infusion was not having a detectable effect on successful task performance.

To allow further analysis of reaching behaviour, high-speed video recordings were obtained from a subset of rats performing the pellet reaching task (*n* = 7; see Section 2.10 for details). The duration of the outward reach towards the pellet was significantly longer following drug infusion compared to saline (Figure 4ci, *t*_6_ = −3.668, *P* = 0.010). However, the endpoint of the reach was not significantly different (Figure 4cii, *t*_6_ = −0.839, *P* = 0.434). The latter suggests that under- or overreaching did not account for the difference in reach duration, in accordance with the similarity in success rate between drug and control groups.

There was a positive correlation between average reach duration and average endpoint of reach for each animal (saline: *r*_5_ = 0.751, *P* = 0.052; drug: *r*_5_ = 0.889, *P* = 0.007). As might be expected, those rats that performed longer distance reaches generally took more time to perform them (Figure 4ciii). However, this relationship was shifted towards increased reach durations for the drug infusion group, with no significant change in end-point of reach (Figure 4ciii), indicating slower reaches. Together, these results therefore provide evidence that following infusion of a mix of muscarinic and nicotinic antagonists into the interpositus nuclei, rats tend to perform reaches more slowly, but the drug infusion does not affect their ability to successfully retrieve reward pellets.

### 3.5 Infusion of cholinergic antagonists into the interpositus nuclei reduces reward pellet consumption

**FIGURE 5:**
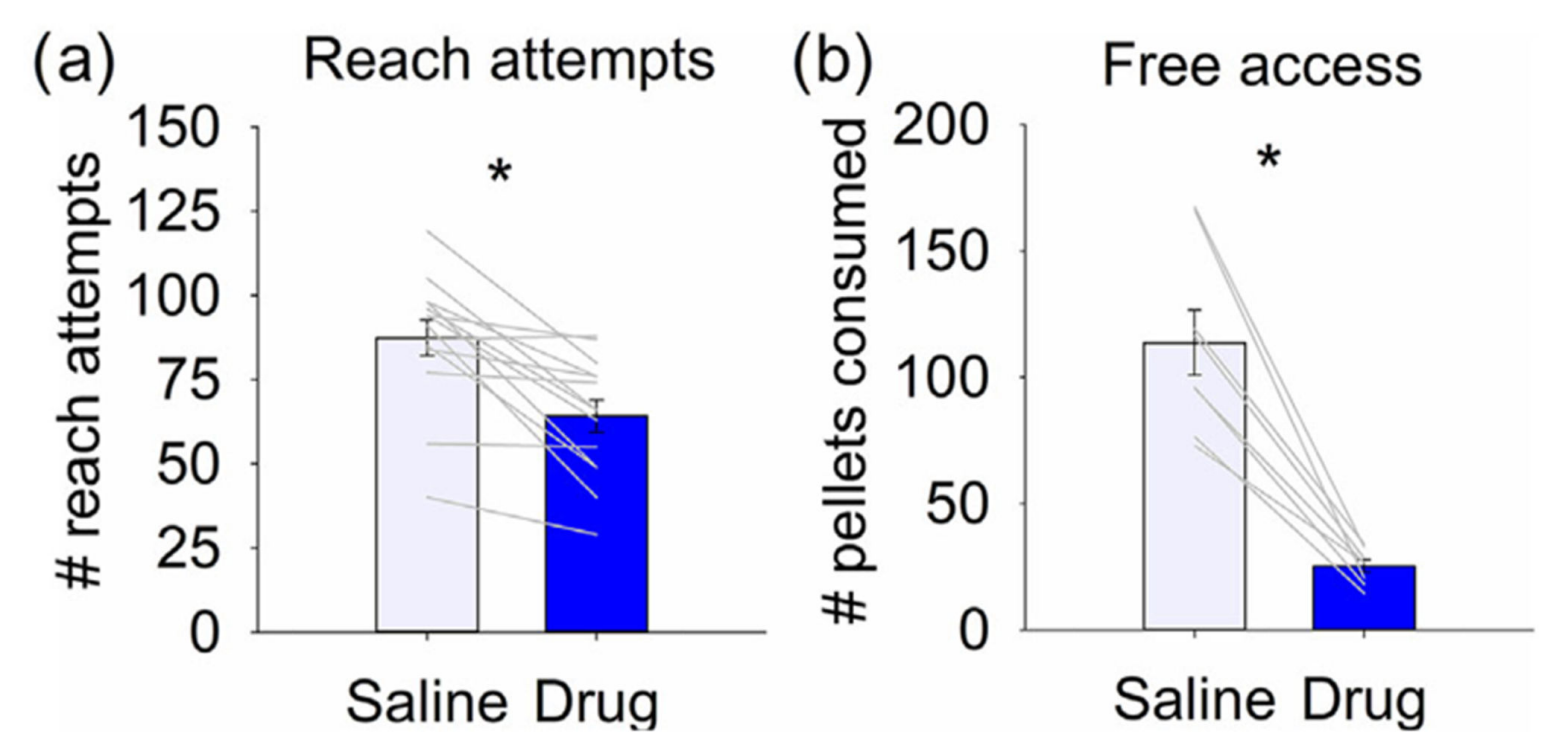
Infusion of cholinergic antagonists into the interpositus nuclei of the cerebellum reduced the number of reward pellets consumed. (a) Number of self-initiated reach attempts in the pellet reaching task following infusion of saline or drug (scopolamine and mecamylamine, *n* = 14). (b) Approximate number of reward pellets consumed when allowed free access (*n* = 8). *P < 0.05.

Although the proportion of successful reaches was not affected by the infusion of cholinergic antagonists into the interpositus nuclei (Figure 4), rats performed significantly fewer reaches following drug infusion compared to saline (Figure 5a, *t*_13_ = 4.769, *P* < 0.001, *n* = 14). One possible reason for fewer reaches is a drug-induced reduction in appetite. This may also have influenced performance in the other tasks that involved food reward (e.g. the beam walking task). Rats were therefore also assessed in an arena where there was free access to reward pellets. Rats with the drug infusion consumed significantly fewer pellets compared to the saline control group (Figure 5b, *t*_7_ = 7.232, *P* < 0.001, *n* = 8).

These data therefore suggest that there may be some motivational or appetite change following infusion of a mix of muscarinic and nicotinic antagonists into the interpositus nuclei and/or that there could be some physical difficulty in consuming food rewards that is independent of reaching behaviour. In relation to the latter possibility, careful inspection of the animals in the pellet reaching task (captured in video recordings with a clear side-profile view of the rats) indicated no obvious difficulty in the ability to eat. This suggests a motor deficit in feeding behaviour was not the main reason for the difference in pellet consumption. However, more subtle effects on eating and swallowing cannot be excluded. Also, given that the reaching task involves consuming only one pellet at a time, other issues may arise when multiple pellets are eaten in quick succession, as may occur in the free access experiment.

### 3.6 Infusion of cholinergic antagonists into the interpositus nuclei does not impair motor learning

**FIGURE 6:**
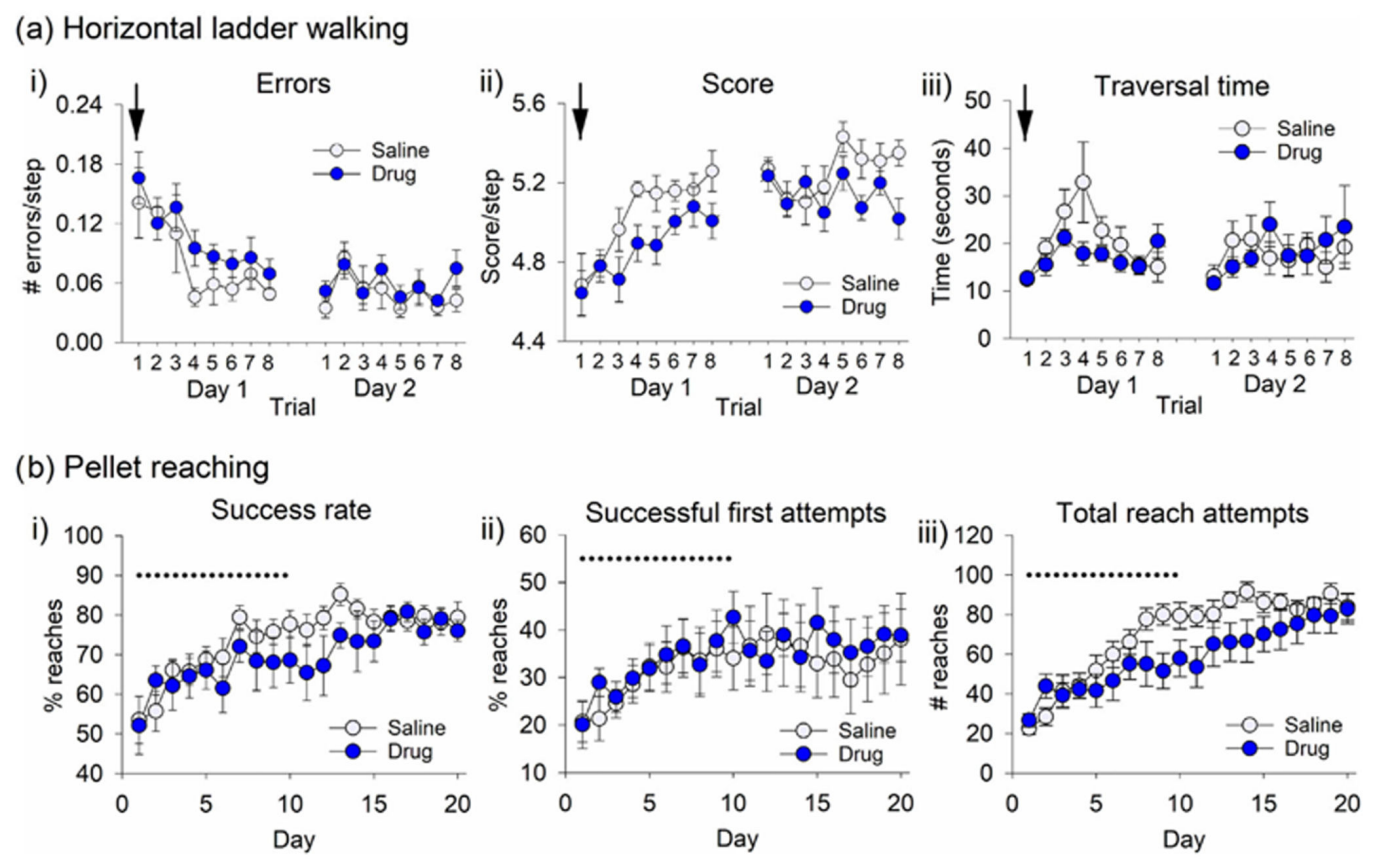
Infusion of cholinergic antagonists into the interpositus nuclei of the cerebellum caused no impairments in motor learning. (a) Rats were infused with either saline or drug (scopolamine and mecamylamine, arrow) and then presented with a novel rung configuration on the ladder walking task for eight trials on Day 1. Rats were tested on a further eight trials the following day (Day 2), with no infusions. (i) Average number of errors per step, (ii) average score per step and (iii) traversal time were recorded (*n* = 9 per group). There was a significant effect of trial on Day 1 for all measures; a significant effect of treatment group was only observed for score on Day 1 (*P* = 0.038). (b) Rats were trained on the pellet reaching task for 10 min per day and infused with either saline or drug following training for 10 days (black dotted line); after this, performance without infusions was assessed for a further 10 days. (i) Success rate on any attempt, (ii) success rate on first reach attempt and (iii) total number of reach attempts were recorded (*n* = 11 per group). For all measures, there was a significant effect of training session; only for total reach attempts was there a significant interaction between training session and treatment group (*P* = 0.016).

Given the role of acetylcholine in the forebrain in learning and memory, the possibility was also explored that blocking cerebellar cholinergic signalling affects motor learning. In the first paradigm, rats were infused with drug or saline before being exposed to the horizontal ladder walking task (Figure 3bi) with novel rung spacing for eight trials (*n* = 9 rats per group). The timing of this infusion meant it was likely to impact both motor learning within the session and the early consolidation period following the session. In the initial learning phase (Day 1), there was a significant effect of trial on the number of errors (Figure 6ai, *F*_7,122_ = 8.134, ϵ = 0.453, *P* < 0.001), score (Figure 6aii, *F*_7, 112_ = 8.665, *P* < 0.001) and traversal time (Figure 6aiii, *F*_7,122_ = 4.836, ϵ = 0.371, *P* = 0.008), confirming that with experience, both groups learned to adapt to the novel rung configuration and improved their performance after repeated trials.

During the learning phase (Day 1), there was a significant effect of treatment on average step score (Figure 6aii, *F*_1, 16_ = 5.143, *P* = 0.038); however, the only statistically significant pairwise difference between the saline and drug groups was on Trial 4 (*t*_16_ = 2.758, *P* = 0.019, corrected as equal variances not assumed). This suggests that there may have been a transient performance deficit in the drug group (as seen in Figure 3b). However, given that start and end performance were similar between groups, learning was not significantly affected overall.

On Day 2, rats were tested for eight trials on the same rung configuration as Day 1, but without infusions, to test how well they had retained the ability to traverse the ladder. There was a significant effect of trial on errors (Figure 6ai, *F*_7,112_ = 2.247, *P* = 0.035) and score (Figure 6aii, Day 2, *F*_7,112_ = 2.374, *P* = 0.028), suggesting some change in performance across trials within a session. However, there were no significant effects of group, or interactions between trial and group, for any of the measures. This suggests that infusion of a mix of muscarinic and nicotinic antagonists during motor learning did not affect the subsequent ability of rats to learn or recall the ability to traverse the ladder with the novel rung configuration.

The effects of cholinergic antagonists on learning of the pellet reaching task were also investigated (*n* = 11 per group). Rather than showing trial-by-trial improvements in a single day as for ladder walking, the pellet reaching task takes a number of days to reach a plateau in performance. To control for any motor effects during task performance, infusions were given after each training session for 10 days. A further 10 training days were subsequently carried out without infusions. There was a significant effect of day on success rate (Figure 6bi, *F*_19,380_ = 8.804, ϵ = 0.248, *P* < 0.001), successful first attempts (Figure 6bii, *F*_19,380_ = 3.195, ϵ = 0.191, *P* = 0.021) and total reach attempts (Figure 6biii, *F*_19,380_ = 31.508, ϵ = 0.245 *P* < 0.001), showing that the performance of the rats improved over days, that is, that motor learning had occurred.

There were no significant effects of treatment, or interaction between day and treatment, for success rate or successful first attempts. Consistent with the standard pellet reaching task described above, there was, however, a significant interaction between day and treatment for total reach attempts (Figure 6biii, *F*_19,380_ = 3.046, ϵ = 0.245, *P* = 0.016), showing the drug group performed fewer reaches than the saline group. The recovery of this difference in the later stages of training (Figure 6biii) suggests that there were no long-term adverse effects of repeated drug infusions.

In summary, the pellet reaching data suggest that the performance of both drug and control groups improved with experience, as assessed by an increasing proportion of successful reaches over time. However, the drug group performed fewer reaches as learning progressed. This suggests that the ability of rats to successfully retrieve pellets was not altered by the mix of cholinergic antagonists but that there was a reduction in the number of self-initiated trials. This may reflect reduced motivation and/or a reduction in spontaneous movements required to trigger new trials. The latter, because rats had to shuttle to the back of the box to break the LED beam to trigger the release of each pellet.

### 3.7 Separate infusion of muscarinic and nicotinic antagonists into the interpositus nuclei

**FIGURE 7:**
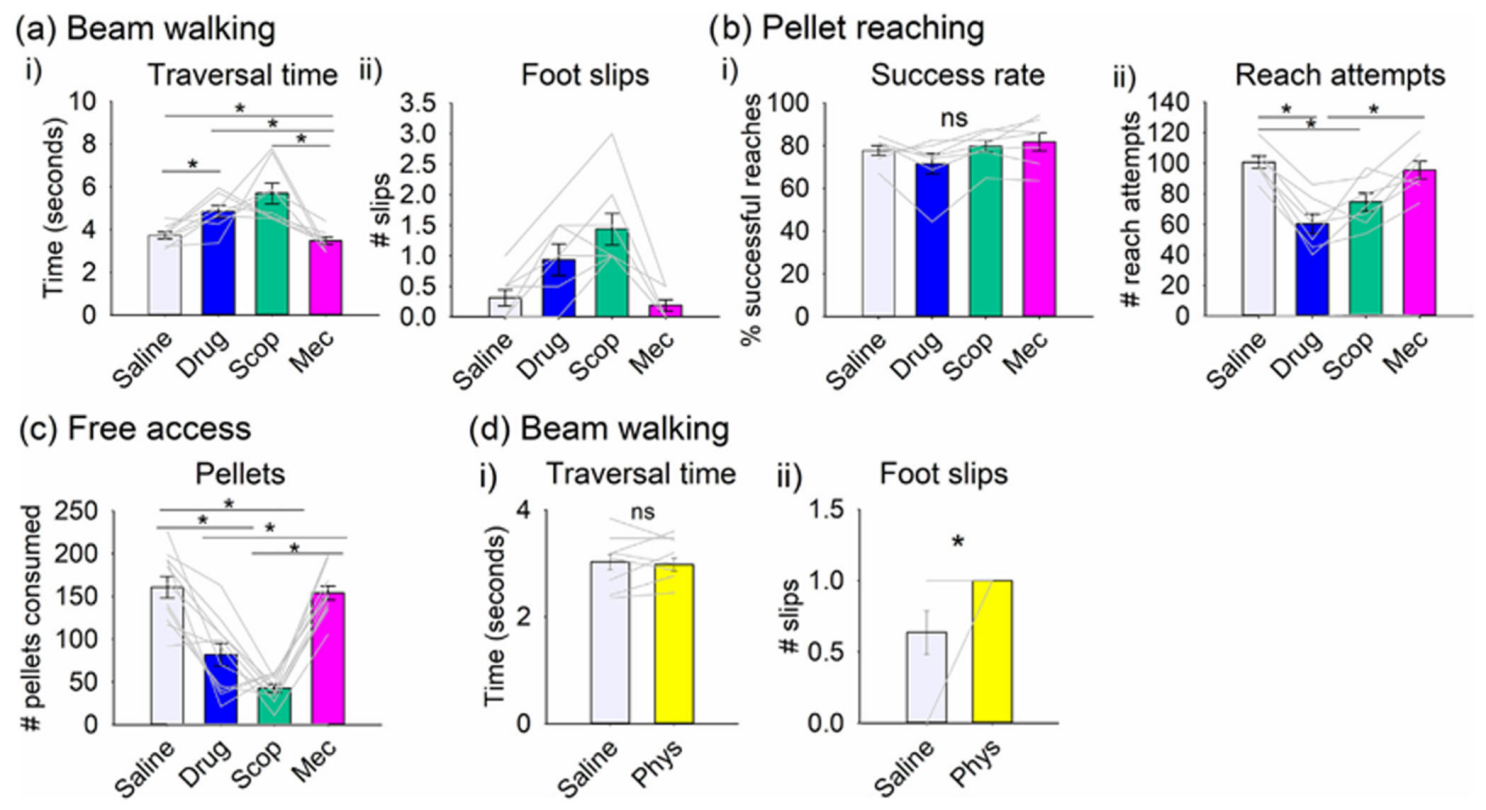
Blockade of muscarinic receptor signalling results in motor performance deficits. The effect of infusion of scopolamine and mecamylamine individually by comparison to a mix of the two drugs was examined in a selection of behaviours. (a) For beam walking (*n* = 8 rats), there was a statistically significant difference between infusions of saline, the combination of cholinergic antagonists (drug) and individual antagonists in relation to (i) traversal time (*P < 0.05) and (ii) number of foot slips (*P < 0.05). (b) For pellet reaching (*n* = 7 rats), there was no statistical difference between treatment groups in relation to (i) success rate of reaching (ns, *P* > 0.05), but (ii) there was a statistically significant difference in number of reach attempts (*P < 0.05). (c) For free access to reward pellets (*n* = 11 rats), there was a statistically significant difference between treatment groups (*P < 0.05). (d) In the beam walking task, there was no statistical difference between infusion of physostigmine (Phys) and vehicle (*n* = 11 rats) in (i) traversal time (ns, *P* > 0.05), but (ii) a statistically significant increase in foot slips (*P < 0.05).

To separate out the roles of muscarinic and nicotinic receptors on motor function, scopolamine and mecamylamine, respectively, were infused individually to observe their effect on a range of behaviours (*n* = 8 rats). For beam walking, there was a statistically significant difference in traversal time between infusions of saline, the drug combination, scopolamine and mecamylamine (Figure 7ai, *F*_3,21_ = 9.609, ϵ = 0.045, *P* = 0.009). By comparison to saline, traversal of the beam took a significantly longer time following infusion of the muscarinic antagonist scopolamine (pairwise comparison *P* = 0.006), and there were also significantly more foot slips (Figure 7aii, *F*_3,21_ = 13.234, *P* < 0.001). By contrast, there was a slight but significant decrease in traversal time when the nicotinic antagonist mecamylamine was infused compared to saline (pairwise comparison *P* = 0.026). Overall, these results suggest that rapid and coordinated beam walking is supported by muscarinic signalling in the cerebellar interpositus nuclei.

For pellet reaching (*n* = 7 rats), there was a significant difference in success rate between the four infusion groups (Figure 7bi, *F*_3,18_ = 4.331, *P* = 0.018) but no significant pairwise comparisons (ns). For number of reach attempts, there was a significant difference between the infusions (Figure 7bii, *F*_3,18_ = 23.611, *P* ≤ 0.001). There was a significant reduction in reach attempts between saline and the drug combination (*P* ≤ 0.001) and also between saline and scopolamine (*P* = 0.007), but not between saline and mecamylamine (*P* = 1.00). This suggests that muscarinic receptor signalling in the interpositus nucleus is the key cholinergic contributor to pellet reach attempts.

Rats were also assessed in an arena in which they had free access to reward pellets (*n* = 11 rats). Consistent with the data shown in Figure 5b, rats infused with a mix of cholinergic antagonists consumed significantly fewer pellets compared to the saline control group (*F*_2.1, 21.6_ = 40.5, *P* ≤ 0.001, pairwise comparison *P* = 0.001; Figure 7c). Similarly, scopolamine but not mecamylamine resulted in significantly fewer pellets consumed compared to the saline control group (pairwise comparison *P* ≤ 0.001; Figure 7c). This suggests that muscarinic rather than nicotinic receptor signalling in the cerebellar nuclei is also involved in regulating pellet consumption.

### 3.8 Infusion of physostigmine to enhance physiological acetylcholine levels in the interpositus nuclei

The data so far have assessed physiological roles of muscarinic and/or nicotinic acetylcholine receptors in interpositus nucleus by perfusion of appropriate antagonists. As an alternative means of modulating cholinergic signalling we also used infusion of the cholinesterase inhibitor physostigmine, which enhances cholinergic signalling by prolonging the activity of endogenously released acetylcholine. Rats (*n* = 11) received an infusion of physostigmine or vehicle before carrying out the beam walking task. Physostigmine had no detectable effect on beam traversal time (Figure 7di) but significantly increased the number of foot slips (Figure 7dii, t_10_ = 2.39, *P* = 0.037). The increase in foot slips resembled the effect of the muscarinic antagonist scopolamine (Figure 7aii) and a possible explanation for this is considered in Section 4.

## 4 Discussion

The current experiments provide evidence that, in rats, blocking cholinergic signalling in the cerebellum, centred on the interpositus nucleus, results in behavioural deficits including a reduction in spontaneous locomotion and rearing behaviour, impaired coordination of locomotion, slower performance of skilled movements and a decrease in the consumption of food rewards. These effects appear to be associated mainly with disruption of muscarinic receptor signalling within the cerebellum. Given that the interpositus nucleus is the outflow of the paravermal cerebellum, physiological release of acetylcholine is likely to be important for limb movements through increasing speed and precision, and possibly through increasing motivation for reward. By contrast, no significant role of acetylcholine in the interpositus nuclei was detected in our experiments for motor learning in the ladder walking or pellet reaching tasks. This raises the possibility that the role of acetylcholine, at least in the paravermal region of the cerebellum, differs from other brain regions, such as the neocortex and hippocampus where acetylcholine is required for learning processes (Hasselmo, 2006).

Neocortical cholinergic projections arise from the basal forebrain cholinergic nuclei, whereas cholinergic projections to the cerebellar nuclei arise predominantly from brainstem sources, most notably the PPN (Jaarsma et al., 1997; Woolf & Butcher, 1989). There are several disorders involving degeneration of the PPN, including Parkinsonian type multiple system atrophy and progressive supranuclear palsy, in which some of the key symptoms resemble the deficits observed in the current experiments. Namely, difficulty in initiating movements, gait disturbances, slowed movement and poor coordination (Fabbrini et al., 2019; Gilman et al., 2010; MacLaren et al., 2018). Patients may also present with dysphagia (Calandra-Buonaura et al., 2021; Warren et al., 2005), reflecting the changes in feeding behaviour observed here. Our data therefore highlight the possibility that loss of cholinergic projections to the cerebellum may have an important contribution to the impairments observed in such disease states.

### 4.1 Non-uniformity of cholinergic projections to the cerebellar nuclei

Cholinergic projections to the cerebellum are topographically organised, with the highest density of projections within the cerebellar nuclei targeting the lateral nuclei and posterior interpositus nuclei and less dense projections within the anterior interpositus nuclei and medial cerebellar nuclei (Jaarsma et al., 1997; Vitale et al., 2016). In eyeblink conditioning, the anterior interpositus has been shown to be important for learning of the conditioned reflex, while the posterior interpositus plays a role in modulating motor responses but not the learning process (Jiménez-Díaz et al., 2004). Given the topographical organisation of cholinergic projections to anterior versus posterior subdivisions of the interpositus nucleus, and our findings of performance but not learning deficits, our data are consistent with the possibility that the action of the cholinergic antagonists was mainly directed towards the posterior interpositus nucleus. However, our infusion spread analysis does not support this. Alternatively, it is possible that cholinergic projections to interpositus do not play any significant role in the types of learning investigated in this study but instead play a crucial role in the performance of motor behaviours.

Both muscarinic and nicotinic acetylcholine receptors are present within the cerebellum in both the cortex and nuclei (Jaarsma et al., 1997; Turner et al., 2011). Given that the primary effects observed in our experiments appear to be mediated by muscarinic receptor antagonism, it is important to consider muscarinic receptor distribution within the cerebellum. Within the cerebellar nuclei, muscarinic receptor expression follows the pattern of cholinergic inputs, with the highest density of receptors in the lateral and interpositus nuclei (Beitz et al., 1984). However, the localisation of muscarinic receptors on particular cell types or compartments is unknown.

While some physiological studies have shown functional roles of M1 and M3 muscarinic receptor subtypes in the cerebellum (Pickford et al., 2019; Rinaldo & Hansel, 2013), anatomical studies do not show a significant number, if any, of these receptor subtypes. In contrast, the vast majority of cerebellar muscarinic receptors are of the M2 subtype (Jaarsma et al., 1997; Li et al., 1991; Tice et al., 1996). Given that their cellular localisation in the cerebellar nuclei is not known, it is difficult to predict the mechanisms by which M2 receptors could influence behaviour. However, since M2 receptors are generally presynaptic auto-receptors, they most likely function to regulate acetylcholine release from extracerebellar cholinergic projections, such as those arising from PPN. Therefore, presynaptic M2 receptor blockade by scopolamine may disrupt motor functions by aberrant enhancement of acetylcholine levels. In accord with this, we also show that enhancing acetylcholine levels using physostigmine results in similar deficits in motor function. In the cerebellar cortex, muscarinic binding sites are most dense in the granular and Purkinje cell layers (Neustadt et al., 1988). As our infusions were not confined solely to the interpositus nucleus, it is possible that some of the effects we observed were mediated by disruption of cholinergic signalling in the cerebellar cortex.

### 4.2 Direct versus indirect motor impairments

In rodents, it is difficult to dissociate motor from non-motor functions such as motivation and attention, given they are inextricably linked, and all are determined experimentally by motor actions of an animal. However, given that the impairments we found were evident in both food rewarded (beam walking, pellet reaching) and non-food rewarded tasks (open field, ladder walking), it seems unlikely that changes in motivation for food reward fully accounted for the motor deficits observed. Nonetheless, reward pellet consumption was clearly impacted by infusion of the cholinergic antagonists. There are several possible (not mutually exclusive) reasons for this, including deficits in motivation; motor deficits relating to the act of eating, for example difficulty swallowing; autonomic effects, such as a dry mouth causing difficulty in consuming rewards (known side effect of the antagonists; Spinks & Wasiak, 2011; Nickell et al., 2013); and/or the drugs causing a general malaise.

Effects on the ability to eat and animals feeling unwell could lead to slower performance and eating less. However, we consider this unlikely to fully explain our findings for several reasons. Firstly, systemic administration of scopolamine is used to prevent nausea, which is the opposite to what might be expected if the drug treatment was making the animals unwell (Spinks & Wasiak, 2011). Secondly, our drugs were administered locally within the cerebellum and equate to much lower doses than typically given systemically, minimising off-target effects. Indeed, systemic scopolamine has been shown to increase motor activity in rodents (Wolthuis et al., 1975), the opposite of our finding here. Also, any non-specific effects might be expected to impair motor learning, but this was not the case. Thirdly, animals showed no adverse signs in their home cage and maintained a healthy weight throughout the experiment. And finally, motor coordination was clearly influenced in our experiments, in the form of increased stepping errors on the beam and ladder walking tasks. This effect was most evident after localised infusion of scopolamine into the cerebellum. By contrast, previous studies that have infused scopolamine into other regions of the brain (e.g. perirhinal cortex) found no evidence of an effect on motor performance (Warburton et al., 2003).

### 4.3 Acetylcholine influences speed of information processing

The common theme that emerges from the present study is that blocking cholinergic signalling in the interpositus nuclei of the cerebellum reduces the occurrence and/or speed of voluntary behaviour across a range of behavioural tests (open field, rearing, beam walking and pellet reaching). Movements were usually slower and occurred less frequently. This suggests that one role of acetylcholine in the cerebellum may be to promote appropriate and efficient movements. Broadly consistent with this interpretation are the findings in humans that cholinergic signalling influences the speed of information processing when assessing both individual reaction times (Schneider et al., 2015) and the speed of information processing independent of motor responses (Hutchison et al., 2001).

In the cerebral cortex, the ability of acetylcholine to refine information processing may be through its effects on enhancing behaviourally relevant signals while reducing background noise levels (Minces et al., 2017). A similar mechanism may occur in the cerebellum to support more efficient motor behaviour. More specifically, the blockade of cholinergic signalling in the current experiments may have impaired the ability to discriminate task-specific from background sensorimotor signals, leading to an impaired ability to execute motor behaviours with appropriate speed and accuracy.

## 5 Conclusion

The current study provides evidence for a role of acetylcholine in the cerebellum in modulating motor behaviour mediated mainly via muscarinic receptor signalling. Intact cerebellar cholinergic signalling may improve motor performance by supporting spontaneous movement, precision of stepping, speed of execution of locomotion and reaching and possibly by increasing appetite for food reward. The central pathway(s) involved remain to be determined, for example, by using cell specific viral targeting methods. A key candidate is the cholinergic PPN–cerebellar projection, and we are currently exploring this possibility. The present results therefore have implications for diseases involving degeneration of the brainstem cholinergic system, adding the cerebellum as a potential target for treatment of cholinergic dysfunction.

## Figures and Tables

**TABLE 1:** Scoring for ladder walking task (Metz & Whishaw, 2002).

Score	Type of placement	Description
0	Total slip	Rung missed and fall occurred
1	Deep slip	Fall after limb slipped from rung
2	Slight slip	Slight fall after limb slipped from rung
3	Replacement	Limb replaced from one rung to another
4	Correction	Limb aimed for one rung but placed on another; or position corrected on same rung
5	Partial placement	Limb placed on rung with digits/toes or wrist/heel
6	Correct placement	Midportion of limb placed on rung

## Data Availability

The data that support the findings of this study will be made available upon request.
